# Impact of acute kidney injury in ≥65-year-old kidney donors on short- and long-term allograft outcomes

**DOI:** 10.3389/fmed.2025.1683082

**Published:** 2026-01-21

**Authors:** Quirin Bachmann, Lukas Nebl, Agathe Basta, Florian Kälble, Christoph F. Mahler, Matthias Ott, Matthias C. Braunisch, Volker Assfalg, Uwe Heemann, Jürgen Dippon, Lutz Renders, Vedat Schwenger, Fabian Echterdiek

**Affiliations:** 1 Department of Nephrology, University Hospital Rechts der Isar, Technical University of Munich, TUM School of Medicine and Health, Munich, Germany; 2 Department of Nephrology, Heidelberg University Hospital, Heidelberg, Germany; 3 Department of Emergency and Intensive Care Medicine, Klinikum Stuttgart –Katharinenhospital, Stuttgart, Germany; 4 Department of Surgery, University Hospital Rechts der Isar, Technical University of Munich, TUM School of Medicine and Health, Munich, Germany; 5 Institute for Stochastics and Applications, University of Stuttgart, Stuttgart, Germany; 6 Department of Nephrology, Klinikum Stuttgart – Katharinenhospital, Stuttgart, Germany

**Keywords:** acute kidney injury, death-censored graft survival, delayed graft function, donor age, Eurotransplant Senior Program, kidney transplantation

## Abstract

**Background:**

Kidney transplantation (KT) from elderly donors (aged ≥65 years) with acute kidney injury (AKI) remains controversial and these organs might be underutilized. To date, clear evidence supporting the safety of KT from donors with AKI exists solely for younger donor populations. We hypothesized that, when appropriately selected, graft survival and function in recipients of kidneys from AKI and non-AKI donors aged ≥65 years are comparable.

**Methods:**

We conducted a retrospective cohort study analyzing KT outcomes from donors aged ≥65 years with and without AKI that were performed between 2006 and 2021 at three German transplant centers. AKI was defined according to KDIGO criteria. Death-censored graft survival, overall graft survival, patient survival up to 7 years, eGFR up to 5 years as well as incidence of delayed graft function and biopsy proven acute rejection were compared. Kaplan-Meier analyses and multivariable Cox regression were performed.

**Results:**

Of 685 KT recipients, 183 received kidneys from AKI donors, and 502 from non-AKI donors. Most KTs were from donors with KDIGO stage 1 AKI (*n* = 151; 81.6%). Delayed graft function occurred similarly often in AKI and non-AKI recipients (32.8% vs. 32.8%, *p* = 1.0). Death-censored graft survival was comparable between AKI and non-AKI groups (7 years: 59.0% vs. 61.3%; *p* = 0.87). Median eGFR at 12 months was 33.8 mL/min/1.73 m^2^ (IQR 27.3, 44.2) in the AKI group and 35.5 mL/min/1.73 m^2^ (IQR 26.3, 44.8) in the non-AKI group (*p* = 0.79). These results remained unchanged after adjustment for known risk factors of graft survival in the multivariable Cox regression.

**Conclusion:**

In this study, KT from ≥65-year-old donors with mostly mild AKI resulted in similar short and long-term graft survival and function compared to KT from ≥65-year-old donors without AKI. These findings support the utilization of AKI kidneys from elderly donors to expand the donor pool without compromising outcomes.

## 1 Introduction

Due to the universal donor kidney shortage, organs previously deemed unsuitable because of advanced donor age or pre-existing medical conditions are increasingly being utilized (1–3). In the Eurotransplant (ET) region, 29% of all deceased donor kidney transplants in 2023 were procured from donors aged 65 years or older and the number of expanded criteria donors (ECD) has increased to about 45% (4, 5). Growing evidence demonstrates a survival benefit for patients receiving transplants from older donors compared to patients remaining on maintenance dialysis (6–9). However, especially kidneys from ≥65-year-old donors are particularly vulnerable due to the higher prevalence of comorbidities, such as diabetes mellitus, and due to the higher susceptibility to prolonged cold ischemia times (CIT) and the development of delayed graft function (DGF)—all of which are known to impair allograft survival (10–12). These circumstances make kidney transplantation (KT) with grafts from ≥65-year-old deceased donors challenging.

About 15–25% of deceased donors are additionally affected by an acute kidney injury (AKI) before organ procurement which further increases the risk for DGF and has led to high discard rates (13–15). Whether kidneys from ≥65-year-old donors with AKI can be transplanted with satisfactory outcomes is largely unknown. Preliminary single-center data from our group previously suggested no significant decrease in allograft survival and function (16). However, large-scale multicenter studies are required to better guide management decisions in this challenging group of high-risk AKI donors. Thus, we performed a multicenter retrospective study to analyze outcomes of KTs from ≥65-year-old donors with and without AKI.

## 2 Materials and methods

### 2.1 Study population

This study included all deceased donor KTs from donors aged ≥65 years that were performed between 01/01/2006 and 31/12/2021 at three transplant centers in Germany: Heidelberg University Hospital (*n* = 308), Technical University of Munich University Hospital (*n* = 175) and Klinikum Stuttgart (*n* = 202). Since donations after cardiac death are prohibited in Germany, all KTs were donations after brain death. All organs were kept in static cold storage before implantation. Combined organ transplantations were excluded from the study. The total number of KTs (*n* = 685) is higher than the total number of KT donors (*n* = 578), as both kidneys from one donor were transplanted in 18.5% of all donors in which case both KTs were included in the analysis.

AKI was classified according to the 2012 KDIGO Clinical Practice Guideline for Acute Kidney Injury (17). As information on urinary output of donors was not available over the full course of the intensive care unit stay, urinary output was not used to grade AKI. Additionally, because true baseline creatinine levels are typically unavailable for deceased kidney donors, increases in serum creatinine were calculated based on the difference between creatinine levels at hospital admission and creatinine levels at organ recovery: AKI stage 1, serum creatinine at organ recovery 1.5–1.9 times elevated compared to admission creatinine or an absolute increase of ≥26.5 μmol/L; stage 2, serum creatinine at organ recovery 2.0–2.9 times elevated compared to admission creatinine; stage 3, serum creatinine at organ recovery ≥3 times elevated compared to admission creatinine, or an absolute increase to ≥353.6 μmol/L, or initiation of renal replacement therapy (18). For secondary analysis, a stratification according to AKI severity was performed. As donors with stage 2 (moderate) and stage 3 (severe) AKI constituted only 16.2% of the study population, these two stages were combined for this AKI-stratified analysis. Additionally, all donors with AKI (stages 1–3) were also categorized into “resolving” AKI, defined as having a creatinine value at organ recovery that was ≥0.3 mg/dl lower than the peak creatinine value and “ongoing” AKI, if this was not the case. The kidney donor risk index (KDRI) was calculated from donor characteristics according to the formula of the Organ Procurement and Transplantation Network (OPTN) (19). Cases with missing information about hypertension or diabetes were excluded from the analysis. Using the OPTN mapping table with the scaling factor of 2024, the KDRI was translated into the KDPI score (%).

Induction therapy was routinely performed with an interleukin-2 receptor antibody (Basiliximab). Recipients with higher risk for rejection, e.g. patients receiving a second kidney transplant were treated with anti-thymocyte globulin (ATG) based on physicians' decision. Baseline immunosuppression after KT consisted of a glucocorticoid (prednisone), calcineurin inhibitors (tacrolimus or cyclosporine A) and mycophenolate mofetil.

### 2.2 Data collection

Donor data were extracted from the ET database in Leiden, The Netherlands. Recipient and kidney-transplant-related data were collected from the electronical hospital information system of each participating center and the ET database. Recipient follow-up was completed with data from local care-giving nephrologists when necessary. This retrospective analysis was approved by the local ethics committees at Heidelberg (S-187/2022), Munich (2023-313-S-KH) and Tübingen (632/2019BO2).

### 2.3 Endpoints

The primary endpoints were defined as death-censored graft survival, overall graft survival and patient survival at 7 years after KT. All causes of graft loss, including patient death with a functioning graft, were considered when calculating overall graft survival. Patient survival was defined as overall survival irrespective of graft function, including patients who returned to dialysis. Secondary endpoints included length of hospital stay during kidney transplantation, eGFR 3 months, 1, 3, and 5 years after KT as well as incidence of DGF, primary non-function (PNF), and incidence of biopsy proven acute rejection (BPAR) within the first three years after KT. BPAR was classified according to the respective Banff classification at the time of biopsy. For the purposes of this analysis, all possible kidney allograft rejection types (acute and chronic T-cell and antibody-mediated rejections) as well as borderline rejections were scored as BPAR (20). The Chronic Kidney Disease Epidemiology Collaboration (CKD-EPI) formula was used to calculate eGFR (ml/min/1.73 m^2^) from recipients' creatinine values (21). DGF was defined as need for at least one dialysis during the first week after transplantation.

### 2.4 Statistics

Descriptive statistical analysis summarized numerical data using the median and interquartile range (IQR), while categorical data were expressed as percentages. Kruskal-Wallis rank sum test assessed whether numerical samples originated from the same distribution, and Fisher's exact test evaluated the independence of two categorical variables.

For right-censored longitudinal data, survival rates were estimated using the Kaplan-Meier method, and group comparisons were performed with the log-rank test. Cox regression analysis was performed to assess the influence of potential risk factors while adjusting for confounders, incorporating two random effects to account for possible transplant center variability and for potential dependencies introduced by kidneys from the same donor. In these Cox regression models, we considered the donor-related variables age, sex, BMI, cause of death, diabetes, and arterial hypertension, along with the recipient-related variables age, sex, BMI, duration of dialysis, diabetes, arterial hypertension, highest PRA and number of KTs as well as the transplant-related variables number of HLA mismatches and cold ischemia time as potential risk and confounding factors. The objective of the multivariable risk models was not survival prediction, but an unbiased and precise estimation of the causal effect of AKI stage on overall graft, death-censored graft and patient survival under standard allocation conditions. Accordingly, all previously specified variables were retained in the model. The proportional hazards assumption was routinely examined. Missing values in high-dimensional models were handled through multiple imputations.

All statistical analyses were conducted in R (version 4.5.1) (22), utilizing specialized libraries for survival analysis (23), mixed-effects modeling (24), and multiple imputation (25).

## 3 Results

In total, this study included 685 KTs from 578 deceased, braindead kidney donors. Of these, 183 KTs (26.7%) originated from 149 donors with a history of AKI. 125 donors (83.9%) had mild AKI (defined as KDIGO-stage 1), whereas 24 donors (16.1%) had moderate/severe AKI (defined as KDIGO stage 2 or 3). This translated into 151 KTs from donors with mild AKI and 32 KTs from donors with moderate/severe AKI. The remaining 502 KTs performed in our centers were from 429 donors without AKI prior to kidney recovery.

Donor characteristics are shown in Table 1. Donors without AKI were slightly older than donors with AKI (72 years vs. 70 years, *p* = 0.01) There were no significant differences regarding donor sex, history of arterial hypertension, diabetes or smoking as well as cause of death between the two groups. On admission, eGFR did not differ significantly between donors with and without AKI (no-AKI: 83.8 ml/min/1.73 m^2^ vs. AKI: 78.0 ml/min/1.73 m^2^, *p* = 0.11). As expected, both lowest eGFR (no-AKI: 76.5 ml/min/1.73 m^2^ vs. AKI: 47.4 ml/min/1.73 m^2^, *p* < 0.001) and final eGFR before procurement (no-AKI: 86.1 ml/min/1.73 m^2^ vs. AKI: 55.2 ml/min/1.73 m^2^, *p* < 0.001) were significantly lower among donors with AKI. Diuresis during the last 24 h prior to procurement was not different (no-AKI: 3.4 l vs. AKI: 3.7 l *p* = 0.65). No donor was oligouric (diuresis < 500 ml in the last 24 h), anuric or on dialysis prior to organ recovery. The median kidney donor profile index (KDPI) was significantly higher in donors with AKI compared to donors without AKI (81.0 vs. 75.0, *p* < 0.01).

**Table 1:** Donor characteristics of kidney transplants with and without donor AKI.

**Characteristic**	**All donors (*n* = 578)**	**No AKI (*n* = 429)**	**AKI (*n* = 149)**	***p*-value**
Age (years), median (IQR)	72.0 (68.0, 76.0)	72.0 (68.0, 77.0)	70.0 (68.0, 74.0)	0.01^*^
Male sex	277 (47.9)	201 (46.9)	76 (51.0)	0.45
BMI, median (IQR)	26.2 (24.2, 28.7)	26.2 (24.2, 28.3)	26.3 (24.6, 29.4)	0.17
Arterial hypertension	351 (66.1)	260 (65.7)	91 (67.4)	0.75
Diabetes	96 (19.1)	69 (18.4)	27 (21.3)	0.51
Smoking	129 (25.0)	90 (23.7)	39 (28.7)	0.25
Cause of death				0.19
Stroke	78 (13.5)	59 (13.8)	19 (12.8)	
Intracranial bleeding	332 (57.4)	253 (59.0)	79 (53.0)	
Trauma	56 (9.7)	43 (10.0)	13 (8.7)	
Other (e.g. anoxic brain damage)	112 (19.4)	74 (17.3)	38 (25.5)	
eGFR (ml/min/1,73 m^2^)				
On admission, median (IQR)	82.7 (66.4, 91.0)	83.8 (67.4, 90.9)	78.0 (58.4, 91.0)	0.11
Lowest, median (IQR)	70.0 (56.1, 86.8)	76.5 (64.0, 88.7)	47.9 (37.0, 59.2)	< 0.001^*^
Final, median (IQR)	79.2 (60.1, 92.0)	86.0 (69.9, 93.0)	55.3 (42.0, 69.0)	< 0.001^*^
Diuresis in last 24 h prior to organ donation (L), median (IQR)	3.5 (2.4, 4.8)	3.4 (2.4, 4.8)	3.7 (2.4; 4.8)	0.65
AKI severity				–
Stage 1	–	–	125 (83.9)	
Stage 2 + 3	–	–	24 (16.1)	
Ongoing AKI at kidney recovery	–	–	114 (76.5)	–
KDPI, median (IQR)	76.0 (63.0, 88.0)	75.0 (62.0, 87.0)	81.0 (68.0, 91.5)	< 0.01^*^

Numbers in brackets represent percentages if not indicated otherwise; percentages are based on the number of available cases for each parameter, excluding missing values; AKI, acute kidney injury; IQR, interquartile range; BMI, body mass index; KDPI, kidney donor profile index; ^*^ if *p* < 0.05.

Further donor characteristics, stratified by AKI severity, are shown in Supplementary Table S1. Of note, donors with moderate/severe AKI were slightly younger than donors with mild AKI or without AKI (68 years vs. 71 years vs. 72 years, respectively, *p* = 0.002) and had slightly less urine output in the last 24 h prior to organ recovery (2.7 l vs. 3.4 l vs. 3.8 l, respectively, *p* = 0.05). eGFR on admission was comparable between all the donor groups (no-AKI: 83.8 ml/min/1.73 m^2^, mild AKI: 79.0 ml/min/1.73 m^2^, moderate/severe AKI: 74.1 ml/min/1.73 m^2^, *p* = 0.28) whereas lowest eGFR and final eGFR were lowest in the moderate/severe donor AKI group (lowest eGFR: no-AKI: 76.5 ml/min/1.73 m^2^, mild AKI: 51.3 ml/min/1.73 m^2^, moderate/severe AKI: 24.0 ml/min/1.73 m^2^, *p* < 0.001; final eGFR: no-AKI: 86.0 ml/min/1.73 m^2^, mild AKI: 57.0 ml/min/1.73 m^2^. moderate/severe AKI: 30.6 ml/min/1.73 m^2^, *p* < 0.001).

Recipient and transplant characteristics are illustrated in Table 2. Median recipient age was 67 years in both groups (*p* = 0.48) and median CIT was also comparable (no-AKI 11.2 h vs. AKI 11.0 h, *p* = 0.21). There was no difference in duration of dialysis prior to KT, underlying renal disease, highest panel reactive antibody (PRA) score or number of HLA-mismatches between KTs with and without donor AKI. There was also no significant difference regarding induction or maintenance immunosuppression. More detailed recipient and transplant characteristics, stratified according to donor AKI severity, are displayed in Supplementary Table S2. Cold ischemia time was comparable between KTs from all donor groups (no-AKI 11.2 h vs. mild AKI 10.7 h vs. moderate/severe AKI 11.3 h, *p* = 0.45). However, there were no KTs with CIT ≥ 19 h among donors with moderate/severe AKI whereas they constituted 8.8% of donors among the no AKI group and 9.9% among the mild AKI group, respectively

**Table 2:** Recipient and transplant characteristics of kidney transplants with and without donor AKI.

**Characteristic**	**All KTs (*n* = 685)**	**No AKI (*n* = 502)**	**AKI (*n* = 183)**	***p*-value**
Age (years), median (IQR)	67.0 (65.0, 70.0)	67.0 (65.0, 69.0)	67.0 (65.0, 70.0)	0.48
Male sex	473 (69.1)	355 (70.7)	118 (64.5)	0.14
BMI, median (IQR)	25.8 (23.7, 28.9)	26.0 (23.7, 29.1)	25.4 (23.6, 28.2)	0.12
Arterial hypertension	502 (88.2)	369 (88.5)	133 (87.5)	0.77
Diabetes	138 (24.3)	98 (23.5)	40 (26.3)	0.51
Duration of dialysis (months), median (IQR)	51.0 (31.0, 73.5)	50.0 (30.0, 73.0)	54.0 (33.5, 75.5)	0.40
RRT				0.49
Hemodialysis	611 (89.3)	450 (89.8)	161 (88.0)	
Peritoneal dialysis	73 (10.7)	51 (10.2)	22 (12.0)	
Underlying renal disease				0.70
Diabetic nephropathy	87 (12.7)	61 (12.2)	26 (14.2)	
Hypertensive nephropathy	76 (11.1)	59 (11.8)	17 (9.3)	
Polycystic kidney disease	96 (14.0)	73 (14.5)	23 (12.6)	
Glomerulonephritis	203 (29.6)	142 (28.3)	61 (33.3)	
Other	133 (19.4)	99 (19.7)	34 (18.6)	
Unknown	90 (13.1)	68 (13.5)	22 (12.0)	
Highest PRA				0.44
0%	400 (59.3)	301 (60.7)	99 (55.6)	
>0 to ≤ 20%	194 (28.8)	139 (28.0)	55 (30.9)	
>20%	80 (11.9)	56 (11.3)	24 (13.5)	
Second/third kidney transplant	52 (8.6)	32 (6.4)	20 (10.9)	0.10
HLA mismatches				0.64
0	14 (2.1)	12 (2.4)	2 (1.1)	
1–2	79 (11.6)	59 (11.8)	20 (10.9)	
3–4	322 (47.2)	230 (46.1)	92 (50.3)	
5–6	267 (39.1)	198 (39.7)	69 (37.7)	
Cold ischemia time (h), median (IQR)	11.2 (8.0, 15.5)	11.2 (8.0, 15.9)	11.0 (7.8, 14.1)	0.21
Cold ischemia time >19 h	55 (8.6)	41 (8.8)	14 (8.1)	0.87
Immunosuppression				
IL2-RA/ATG/none	89.3%/7.3%/3.4%	89.9%/6.1%/4.0%	87.6%/10.7%/1.7%	0.05
Tac/CsA/other	54.6%/44.6%/0.7%	55.3%/44.3%/0.4%	52.7%/45.6%/1.6%	0.20
MMF/Aza	99.9%/0.1%	99.8%/0.2%	100.0%/0.0%	1.0
Corticosteroids	99.4%	99.2%	100%	0.58
Year of transplantation, median (IQR)	2012 (2009, 2017)	2012 (2009, 2017)	2013 (2009, 2016)	0.64

Numbers in brackets represent percentages if not indicated otherwise; percentages are based on the number of available cases for each parameter, excluding missing values; AKI, acute kidney injury; IQR, interquartile range; BMI, body mass index; RRT, renal replacement therapy; PRA, panel reactive antibody; HLA, human leukocyte antigen; IL2-RA, interleukin 2-receptor antibody; ATG, anti-thymocyte globulin; Tac, Tacrolimus; CsA, Ciclosporine A; MMF, mycophenolate mofetil; Aza, Azathioprine; ESP, Eurotransplant Senior Program; KTs, kidney transplantations.

**Figure 1:**
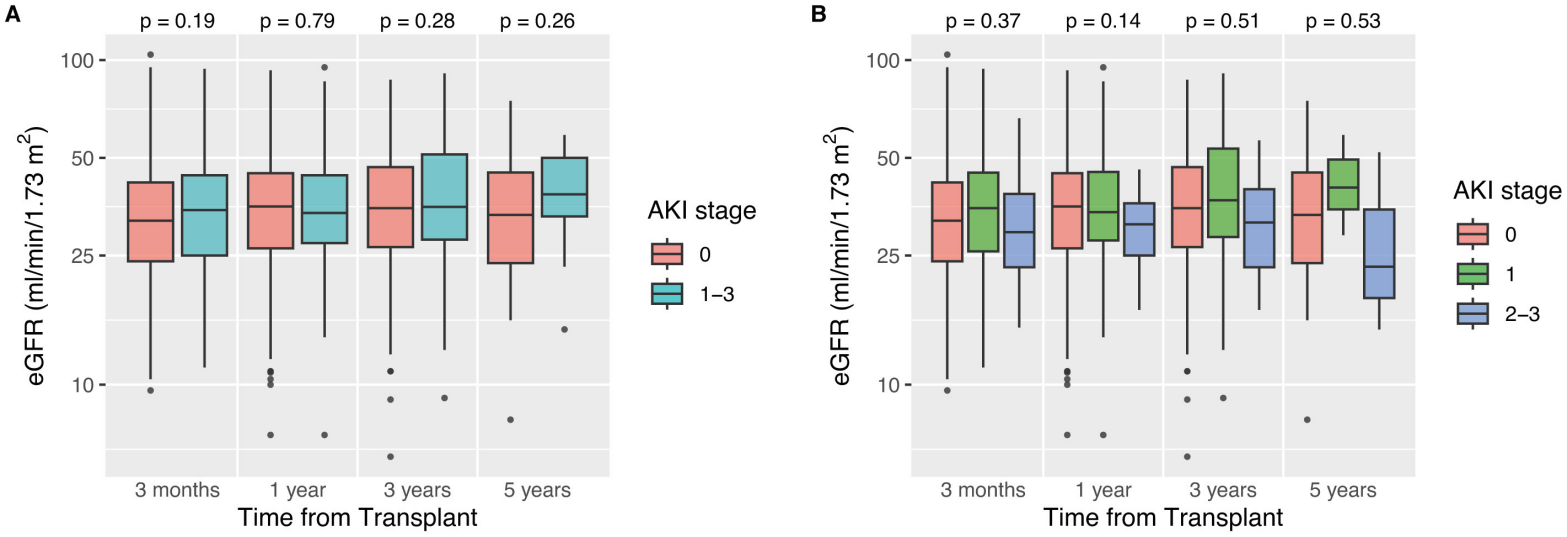
Recipient eGFR (based on CKD-EPI formula) up to 5 years after kidney transplantation from donors with and without acute kidney injury (AKI). **(A)** shows the results for the no-AKI vs. the AKI group. In **(B)** the results for the AKI group were further stratified into mild AKI (stage 1) and moderate/severe AKI (stage 2 + 3). Box-and-whisker plots display the medians and interquartile ranges (IQR) of each sample. The whiskers extend to the smallest and largest values within 1.5 times the IQR. Data points beyond this range are marked with circles and represent outliers. The Kruskal-Wallis chi-squared test was used to assess whether group distributions are equal. eGFR, estimated glomerular filtration rate.

The median follow-up time was 44.0 months for no-AKI KTs and 50.5 months for AKI KTs (*p* = 0.30). DGF occurred in 32.8% of no-AKI KTs and 32.8% of AKI KTs (*p* = 1.0). PNF was also similar between no-AKI and AKI KTs (11.4% vs. 10.4%, *p* = 0.78). The incidence of a first BPAR in year 1–3 after KT as well as the graft function at 3 months, 1 year, 3 years and 5 years after transplant did not differ significantly between KTs from donors with and without AKI (Figure 1A, Table 3). After stratification by AKI severity, we found a higher incidence of DGF among KTs from donors with moderate/severe AKI compared with KTs from donors with mild or no AKI, but these differences did not reach statistical significance (56.2% vs. 40.4% vs. 44.4%, *p* = 0.25; Supplementary Table S3). Similarly, a consistent trend toward lower eGFR at all time points after transplant was observed in KTs from donors with moderate/severe AKI; however, these differences were also not statistically significant (Figure 1B, Supplementary Table S3). The incidence of BPAR also tended to be higher among KTs from donors with moderate/severe AKI but again no significant differences were determined (moderate/severe AKI 53.3% vs. mild AKI 38.2% vs. no-AKI 40.3%, *p* = 0.31). Additionally, Supplementary Table S4 illustrates incidence of BPAR within 3 years after KT depending on the induction therapy.

**Table 3:** Short- and long-term outcomes of kidney transplants with and without donor AKI.

**Characteristic**	**All KTs (*n* = 685)**	**No AKI (*n* = 502)**	**AKI (*n* = 183)**	***p*-value**
DGF	224 (32.8)	164 (32.8)	60 (32.8)	1.0
PNF	76 (11.1)	57 (11.4)	19 (10.4)	0.78
Length of hospital stay (days)	23.0 (16.0, 32.0)	22.0 (16.0, 34.0)	23.0 (16.3, 32.0)	0.81
Recipient eGFR (ml/min/1,73 m^2^), median (IQR)				
3 months after transplant	32.7 (24.4, 43.2)	32.0 (24.0, 42.0)	35.5 (25.0, 44.2)	0.19
1 year after transplant	35.0 (26.4, 44.5)	35.5 (26.3, 44.8)	33.8 (27.3, 44.2)	0.79
3 years after transplant	35.0 (26.9, 47.7)	35.0 (26.6, 46.8)	35.3 (28.0, 51.3)	0.28
5 years after transplant	36.0 (27.7, 48.1)	33.4 (23.7, 45.1)	38.7 (33.0, 50.0)	0.26
BPAR in first year after KT	248 (38.6)	187 (39.3)	61 (36.7)	0.58
BPAR in first three years after KT	261 (40.7)	193 (40.5)	68 (41.0)	0.93
Death-censored graft survival				
1-year	80.5%	80.9%	79.3%	
3-year	73.3%	72.8%	74.4%	
5-year	67.4%	67.6%	66.9%	
7-year	60.6%	61.3%	59.0%	0.87
Overall graft survival				
1-year	74.8%	75.0%	74.2%	
3-year	63.0%	62.0%	65.5%	
5-year	54.8%	54.0%	57.1%	
7-year	44.7%	42.9%	49.4%	0.27
Patient survival				
1-year	88.9%	89.4%	87.3%	
3-year	78.7%	78.3%	80.1%	
5-year	72.8%	72.1%	74.5%	
7-year	62.1%	60.2%	67.4%	0.28
Follow-up time (months), median (IQR)	47.0 (24.0, 86.5)	44.0 (23.0, 86.0)	50.5 (26.0, 93.0)	0.30

Numbers in brackets represent percentages if not indicated otherwise; percentages are based on the number of available cases for each parameter, excluding missing values; graft and- patient survival rates were calculated using the Kaplan-Meier-method; AKI, acute kidney injury; BPAR, biopsy proven acute rejection; DGF, delayed graft function; IQR, interquartile range; KTs, kidney transplantations; PNF, primary non function.

**Figure 2:**
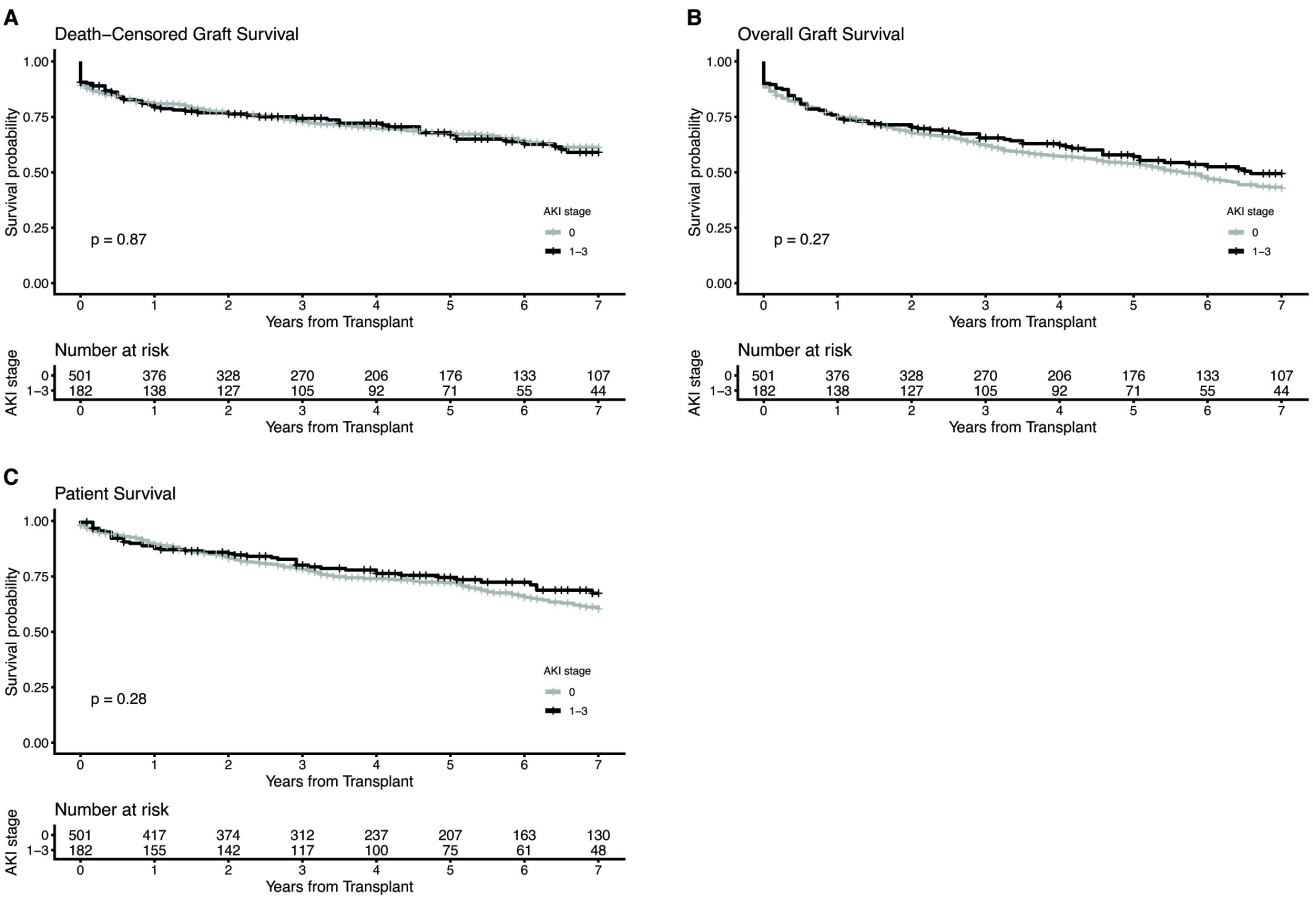
Kaplan-Meier estimates for death-censored graft survival **(A)**, overall graft survival **(B)** and patient survival **(C)** of kidney transplants from donors with and without acute kidney injury (AKI). The log-rank test was used to assess whether group distributions are equal.

**Figure 3:**
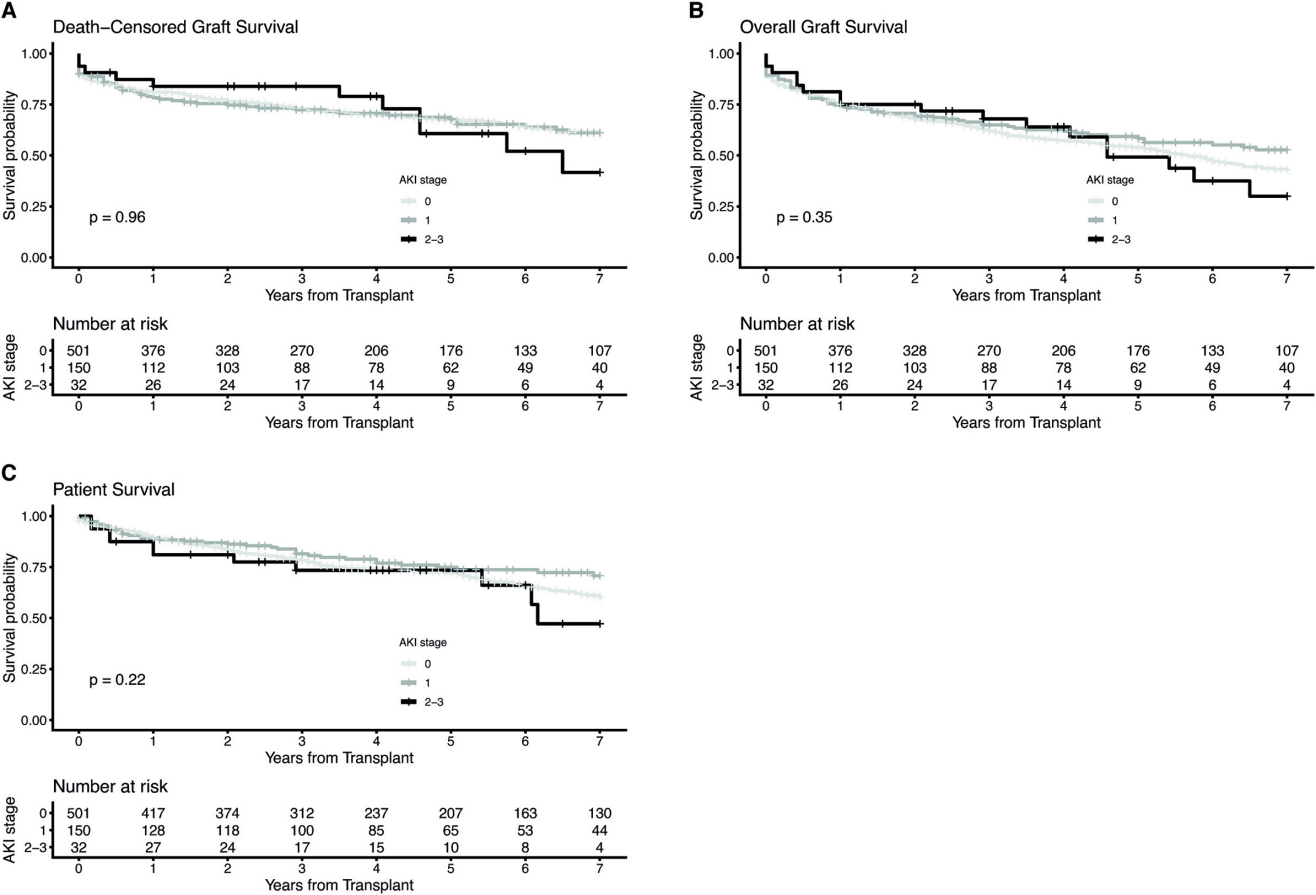
Kaplan-Meier estimates for death-censored graft survival **(A)**, overall graft survival **(B)** and patient survival **(C)** of kidney transplants stratified by severity of donor acute kidney injury (AKI). The log-rank test was used to assess whether group distributions are equal.

7-year death-censored graft survival, overall graft survival and patient survival were similar between KTs from donors with and without AKI (Figures 2A–C, Table 3). After stratification into KTs from donors with mild and moderate/severe AKI, also no differences could be revealed in 7-year death-censored graft survival, overall graft survival or patient survival (Figures 3A–C, Supplementary Table S3). Since HLA-class-II mismatches are a known risk factor for graft loss we further divided no-AKI KTs and AKI KTs by the number of HLA-DR mismatches (Supplementary Table S5). The incidence of BPAR within 1 and 3 years after KT was comparable between AKI and no-AKI KTs within each HLA-DR mismatch group. Moreover, after stratification by the number of HLA-DR mismatches, death-censored graft survival, overall graft survival and patient survival showed no statistically significant differences between AKI and no-AKI KTs.

Finally, we compared KTs from donors with resolving AKI vs. KTs from donors with ongoing AKI at the time of organ recovery. Again, no differences were found in death-censored graft survival, overall graft survival and patient survival (Supplementary Figures S1A–C). Similarly, the incidence of DGF and eGFR up to 5 years after KT were unaffected by whether AKI was ongoing or resolving at the time of organ recovery (Supplementary Table S6).

To further support our findings, a multivariable Cox regression analysis was performed, using a multiple imputation approach and adjusting for known risk factors and confounding factors of death-censored graft loss and death. This analysis also accounted for the influence of different transplant centers and for the impact of KTs from the same donor using a random effects model (Table 4). The results from this analysis confirmed the previous results. AKI was no independent risk factor of death-censored graft loss [hazard ratio (HR) 0.91, 95% confidence interval (CI) 0.65–1.27, *p* = 0.83] or death (HR 0.85, 95% CI 0.60–1.23, *p* = 0.41). A subsequent Cox regression analysis, employing a complete case approach, also confirmed the results (data not shown). We also conducted a multivariable Cox regression analysis after stratification by AKI severity. Both mild as well as moderate/severe AKI were no independent risk factors of death-censored graft loss or death (Supplementary Table S7).

**Table 4:** Fixed effects of multivariable Cox regression of death-censored graft loss and mortality of kidney transplants.

**Covariate**	**Death-censored graft loss**		**Mortality**	
	**Hazard ratio (95% CI)**	***p*** **-value**	**Hazard ratio (95% CI)**	***p*** **-value**
Recipient age	1.02 (0.99–1.04)	0.13	1.07 (1.04–1.11)	< 0.001^*^
Recipient sex: male vs. female	0.89 (0.67–1.19)	0.43	1.27 (0.93–1.76)	0.14
Recipient BMI	1.06 (1.03–1.10)	< 0.001^*^	1.03 (1.00–1.07)	0.08
Duration of dialysis	1.01 (1.00–1.01)	< 0.01^*^	1.00 (1.00–1.01)	0.13
Recipient diabetes: yes vs. no	1.61 (1.14 −2.27)	< 0.01^*^	1.44 (1.02 −2.07)	0.048^*^
Recipient arterial hypertension: yes vs. no	0.74 (0.48–1.14)	0.17	1.16 (0.66–2.02)	0.61
Highest PRA	1.00 (1.00–1.01)	0.42	1.01 (1.00–1.01)	0.18
Number of HLA mismatches	1.04 (0.94–1.15)	0.47	0.97 (0.87–1.08)	0.55
Number of KTs	1.45 (0.85–2.47)	0.17	1.53 (0.89–2.63)	0.13
Cold ischemia time	1.01 (0.98–1.04)	0.50	1.01 (0.98–1.05)	0.37
Donor age	1.00 (0.96–1.04)	0.89	1.02 (0.97–1.07)	0.45
Donor sex: male vs. female	1.03 (0.76–1.39)	0.85	0.74 (0.53–1.04)	0.08
Donor BMI	1.05 (1.00–1.09)	0.03^*^	1.01 (0.96–1.06)	0.72
Donor cause of death cerebral infarction: yes vs. no	1.32 (0.83–2.12)	0.24	0.80 (0.52–1.24)	0.32
Donor arterial hypertension: yes vs. no	0.99 (0.69–1.40)	0.93	1.45 (0.97–2.16)	0.07
Donor diabetes: yes vs. no	0.60 (0.34–1.05)	0.07	1.16 (0.61–1.20)	0.65
Donor KDPI	1.02 (1.00–1.04)	0.57	1.00 (0.97–1.03)	0.98
Donor AKI (any stage): yes vs. no	0.91 (0.65–1.27)	0.83	0.86 (0.60–1.23)	0.41

Missing values of co-variables: Recipient age: 0, Recipient sex: 0, Recipient BMI: 16, Duration of dialysis: 9, Recipient diabetes: 116, Recipient arterial hypertension: 116, Highest PRA: 11, Number of HLA mismatches: 3, Cold ischemia time: 46, Donor age: 0, Donor sex: 0, Donor BMI: 0, Donor cause of death cerebral infarction: 0, Donor arterial hypertension: 56, Donor diabetes: 90, Donor KDPI: 103, Donor AKI: 0.

AKI, acute kidney injury; BMI, body mass index; CI, confidence interval; CVA, cerebrovascular accident; HLA, human leucocyte antigen; KDPI, kidney donor profile index; KTs, kidney transplantations; PRA, panel reactive antibody; ^*^ if *p* < 0.05.

## 4 Discussion

Nowadays, up to 25% of all kidney donors are affected by AKI, often leading to refusal and discard of available grafts despite accumulating evidence indicating that transplantation of AKI kidneys is possible. The evidence is strong for standard criteria donors (SCD) for which a variety of studies, including large scale registry and multicenter studies, have shown that despite increased DGF rates long-term graft survival and function as well as incidence of BPAR are not inferior (13, 26–31). It has also been shown for SCDs that one year after transplantation, kidney grafts from donors with AKI do not display more fibrosis in protocol biopsies than grafts from regular donors (32). However, far less is known about the feasibility of KT from elderly donors with AKI, particularly ≥65-year-old donors even though they already represent more than 25% of all donors in ET countries (33, 34). This provided the rationale for our multicenter analysis.

The results of this study indicate that 7-year death-censored graft, overall graft and patient survival as well as graft function are similar after KT from ≥65-year-old donors with mild AKI compared to those without AKI. Also, we observed no significant differences in 7-year death-censored graft, overall graft or patient survival following KT from donors with moderate or severe AKI compared to non-AKI donors even though a consistent trend to more frequent DGF and lower graft function was found. Importantly, it needs to be stressed that the number of transplants in this subgroup was small (32 KTs from 24 donors), so that these findings should be considered exploratory.

When interpreting our results, it is important to highlight several donor selection criteria within the AKI group that we consider highly relevant for achieving satisfactory transplant outcomes. Importantly, most KTs were from donors with mild AKI and the final eGFR among donors with AKI was only mildly reduced (median 55.3 ml/min/1.73 m^2^). As mentioned above, data on KTs from ≥65-year-old donors with moderate or severe AKI was limited (*n* = 32) which makes meaningful statistical analysis challenging. Of note, although not statistically significant, allograft function was numerically worse at all time points post KT and DGF rates were higher, so that caution regarding ≥65-year-old donors with stage 2 or 3 AKI seems prudent. Moreover, all AKI donors showed normal diuresis (median 3,7 l in the last 24 h prior to explantation) with no donors being oligouric (< 500 ml/24 h), let alone, anuric or on hemodialysis. Also, the CIT among AKI donors was short (median 11.0 h, see section below) and the majority of AKI donors (75%) was less than 75 years old with only 5% aged 80 years or older. These parameters represent crucial donor selection criteria that need to be considered in the context of this study. Thus, transplantation from ≥65-year-old donors with AKI can most likely be considered safe, as long as the AKI stage is only mild, the donor is normouric, < 75 years old, and the expected CIT is < 12 h. Of note, we reported only on KTs from donations after brain death (DBD). We are cautious to extrapolate our findings in this vulnerable, elderly donor population from DBDs to donations after cardiac death because we cannot reasonably estimate how the differences in pathophysiology (e.g. timing of ischemic insult relative to death) will affect outcomes.

The median CIT of 11 h in both groups was significantly lower compared to the median CIT in the standard ET kidney allocation system (ETKAS, ~15 h) (35). This is because in the ET system, kidneys from donors ≥65 years are primarily allocated through the Eurotransplant Senior Program (ESP), which prioritizes short regional transportation times to reduce ischemia injury (36). In our study, more than 80% of KTs were allocated via the ESP. Interestingly, under these circumstances, we did not observe an increased incidence of DGF in KTs from donors with AKI compared to KTs from donors without AKI (32.8% both groups). Nevertheless, the overall incidence of DGF and PNF in this study was rather high. This is likely due to the median donor age of 72 years. It has been shown that grafts from older donors are more vulnerable to CIT, ischemia-reperfusion injury and consequently DGF (11, 12). Previous studies with donors of comparable age have reported similar results (37–41). Importantly, DGF is a known risk factor for BPAR (42, 43). The combination of high DGF incidence and the absence of HLA matching in the ESP (86.3% of KTs with ≥3 mismatches) likely contributed to substantial immune activation, potentially explaining the elevated BPAR rate observed in this cohort.

We previously published data on the effect of AKI in KT from ≥65-year-old donors (16). However, this previous analysis had several shortcomings, as it only included data from one transplant center with a relatively small sample size (*n* = 233). Also, the previous analysis included KTs from a more extensive period (1999–2019) so that not all recipients received a state-of-the art CNI- and mycophenolate-based immunosuppression with additional induction therapy. In contrast, the current analysis comprises data from three transplant centers with almost three times the sample size (*n* = 685) and was limited to the years 2006–2021, i.e. a time-period in which CNI- and mycophenolate-based immunosuppression as well as induction therapy was standard of care which makes the data more relevant to current practice.

Nonetheless, this current study also has several noteworthy limitations. Firstly, compared to nationwide registry studies, the sample size is only moderate. However, kidney transplantation (KT) from donors ≥65 years with AKI is still not widely established. Bearing this in mind, we present a well-sized cohort with detailed clinical characterization of donor, recipient and transplant data. Furthermore, graft and patient survival outcomes were validated through multivariable Cox regression analysis, accounting for other donor, recipient and transplant characteristics and adjusting for the influence of different transplant centers as well as for the impact of KTs from the same donor. Secondly, the retrospective nature of the analysis introduces certain biases. Most importantly, a selection bias must be presumed as we can only report on KTs from donor offers which clinicians in the participating centers deemed suitable for KT in the first place.

In conclusion, we demonstrate that KT from donors aged ≥65 years with mild AKI yields short- and long-term outcomes that are fully comparable to KT from donors without AKI. No significant differences in death-censored graft survival, overall graft survival or patient survival up to seven years post-transplant were observed. Furthermore, graft function remained comparable over time. These findings suggest that concerns about AKI in elderly donors should not preclude organ utilization, especially when the donor has only stage 1 AKI, the expected CIT is < 12 h and the donor is normouric and aged < 75 years. Future prospective studies should specifically address outcomes following KT from elderly donors with moderate and severe AKI, examine associated long-term histological alterations, and contribute to the optimization of allocation strategies to improve transplant success.

## Data Availability

The datasets presented in this article are not readily available because the raw data supporting the conclusions of this article will be made available by the authors upon reasonable request. Requests to access the datasets should be directed to Fabian Echterdiek, f.echterdiek@klinikum-stuttgart.de.

## Supplementary material

Supplementary Figure S1 Kaplan-Meier estimates for death-censored graft survival (A), overall graft survival (B) and patient survival (C) of kidney transplants stratified by recovery status of donor acute kidney injury (AKI). The log-rank test was used to assess whether group distributions are equal.
