# Supplementary material for: Impact of acute kidney injury in ≥65-year-old kidney donors on short- and long-term allograft outcomes
Source: Front Med (Lausanne). 2026 Jan 21;12:1683082. doi: 10.3389/fmed.2025.1683082 (PMC12867811; doi:10.3389/fmed.2025.1683082)
Supplement: Supplementary Figure S1 — Kaplan-Meier estimates for death-censored graft survival (A), overall graft survival (B) and patient survival (C) of kidney transplants stratified by recovery status of donor acute kidney injury (AKI). The log-rank test was used to assess whether group distributions are equal. [file Supplementary_file_1.pdf]

Supplementary Material

Impact of acute kidney injury in ≥65-year-old kidney donors on short- and long-term allograft outcomes

Supplementary Table 1. Donor characteristics of kidney transplants, stratified according to stages of donor AKI.

Characteristic	All donors (n=578)	No AKI (n=429)	AKI stage 1 (n=125)	AKI stage 2+3 (n=24)	p-value
Age (years), median (IQR)	72.0 (68.0, 76.0)	72.0 (68.0, 77.0)	71.8 (68.0, 75.0)	68.0 (67.0, 70.0)	0.002*
Male sex	277 (47.9)	201 (46.9)	64 (51.2)	12 (50.0)	0.66
BMI, median (IQR)	26.2 (24.2, 28.7)	26.2 (24.2, 28.3)	26.3 (24.6, 29.3)	25.7 (24.1, 29.4)	0.30
Arterial hypertension	351 (66.1)	260 (65.7)	76 (67.9)	15 (65.2)	0.91
Diabetes	96 (19.1)	69 (18.4)	25 (23.1)	2 (10.5)	0.41
Smoking	129 (25.0)	90 (23.7)	30 (26.1)	9 (42.9)	0.14
Cause of death					0.67
Stroke	78 (13.5)	59 (13.8)	16 (12.8)	3 (12.5)	
Intracranial bleeding	332 (57.4)	253 (59.0)	66 (52.8)	13 (54.2)	
Trauma	56 (9.7)	43 (10.0)	11 (8.8)	2 (8.3)	
Other (e.g. anoxic brain damage)	112 (19.4)	74 (17.2)	32 (25.6)	6 (25.0)	
eGFR (ml/min/1,73 m²)					
On admission, median (IQR)	82.7 (66.4, 91.0)	83.8 (67.4, 90.9)	79.0 (58.0, 91.1)	74.1 (64.3, 89.2)	0.28
Lowest, median (IQR)	70.0 (56.1, 86.8)	76.5 (64.0, 88.7)	51.3 (42.1, 62.0)	24.0 (19.0, 33.4)	<0.001*
Final, median (IQR)	79.2 (60.1, 92.0)	86.0 (69.9, 93.0)	57.0 (45.3, 73.9)	30.6 (22.1, 40.0)	<0.001*
Diuresis in last 24 hours prior to organ donation (liters), median (IQR)	3.5 (2.4, 4.8)	3.4 (2.4, 4.8)	3.8 (2.5; 5.0)	2.7 (1.8; 3.8)	0.048*
Ongoing AKI at kidney recovery	-	-	96 (76.8)	18 (75.0)	1.0
KDPI	76.0 (63.0, 88.0)	75.0 (62.0, 87.0)	81 (67.0, 93.0)	79 (70.5, 88.0)	0.02*

Numbers in brackets represent percentages if not indicated otherwise; percentages are based on the number of available cases for each parameter, excluding missing values; AKI, acute kidney injury; IQR, interquartile range; BMI, body mass index; KDPI, kidney donor profile index; * if $p < 0.05$.

Supplementary Table 2. Recipient and transplant characteristics of kidney transplants, stratified according to stages of donor AKI.

Characteristic	All KTs (n=685)	No AKI (n=502)	AKI stage 1 (n=151)	AKI stage 2+3 (n=32)	p-value
Age (years), median (IQR)	67.0 (65.0, 70.0)	67.0 (65.0, 69.0)	67.0 (65.0, 70.0)	67.0 (65.0, 69.0)	0.77
Male sex	473 (69.1)	355 (70.7)	93 (61.6)	25 (78.1)	0.05
Arterial hypertension	502 (88.2)	369 (88.5)	110 (88.0)	23 (85.2)	0.80
Diabetes	138 (24.3)	98 (23.5)	32 (25.6)	8 (29.6)	0.65
BMI, median (IQR)	25.8 (23.7, 28.9)	26.0 (23.7, 29.1)	25.4 (23.3, 28.2)	25.5 (24.2, 29.2)	0.23
Duration of dialysis (months), median (IQR)	51.0 (31.0, 73.5)	50.0 (30.0, 73.0)	55.0 (33.0, 76.0)	51.0 (34.3, 63.8)	0.65
RRT					0.54
Hemodialysis	611 (89.3)	450 (89.8)	134 (88.7)	27 (84.4)	
Peritoneal dialysis	73 (10.7)	51 (10.2)	17 (11.3)	5 (15.6)	
Underlying renal disease					0.65
Diabetic nephropathy	87 (12.7)	61 (12.2)	21 (13.9)	5 (15.6)	
Hypertensive nephropathy	76 (11.1)	59 (11.8)	14 (9.4)	3 (9.4)	
Polycystic kidney disease	96 (14.0)	73 (14.5)	19 (12.6)	4 (12.5)	
Glomerulonephritis	203 (29.6)	142 (28.3)	47 (31.1)	14 (43.8)	
Other	133 (19.4)	99 (19.7)	32 (21.2)	2 (6.2)	
Unknown	90 (13.1)	68 (13.5)	18 (11.9)	4 (12.5)	
Highest PRA					0.78
0%	400 (59.3)	301 (60.7)	82 (55.4)	17 (56.7)	
>0 - ≤20%	194 (28.8)	139 (28.0)	46 (31.1)	9 (30.0)	
>20%	80 (11.9)	56 (11.3)	20 (13.5)	4 (13.3)	
Second/third kidney transplant	52 (8.6)	32 (6.4)	14 (9.3)	6 (18.8)	0.06
HLA mismatches					0.89
0	14 (2.1)	12 (2.4)	2 (1.1)	0 (0.0)	
1-2	79 (11.6)	59 (11.8)	17 (11.3)	3 (9.4)	
3-4	322 (47.2)	230 (46.1)	73 (48.3)	19 (59.4)	
5-6	267 (39.1)	198 (39.7)	59 (39.1)	10 (31.2)	
Cold ischemia time (hours), median (IQR)	11.2 (8.0, 15.5)	11.2 (8.0, 15.9)	10.7 (7.5, 14.5)	11.3 (8.1, 13.6)	0.45
Cold ischemia time >19 hours	55 (8.6)	41 (8.8)	14 (9.9)	0 (0)	0.18
Immunosuppression					
IL2-RA/ATG/none	89.3%/7.3%/3.4%	89.9%/6.1%/4.0%	89.1%/9.5%/1.4%	80.6%/16.1%/3.2%	0.07
Tac/CsA/other	54.6%/44.6%/0.7%	55.3%44.3%/0.4%	52.0%/46.0%/2.0%	56.2%/43.8%/0.0%	0.78
MMF/Aza	99.9%/0.1%	99.8%/0.2%	100.0%/0.0%	100.0%/0.0%	1.0
Corticosteroids	99.4%	99.2%	100%	100%	0.64
Year of transplantation, median (IQR)	2012 (2009, 2017)	2012 (2009, 2017)	2012 (2009, 2016)	2014 (2011, 2017)	0.46

Numbers in brackets represent percentages if not indicated otherwise; percentages are based on the number of available cases for each parameter, excluding missing values; AKI, acute kidney injury; IQR, interquartile range; BMI, body mass index; RRT, renal replacement therapy; HLA, human leukocyte antigen; PRA, panel reactive antibody; SD, standard deviation, IL2-RA, interleukin 2-receptor antibody; ATG, antithymocyte globulin; Tac, tacrolimus; CsA, Ciclosporine A; MMF, mycophenolate mofetil; Aza, azathioprine; ESP, Eurotransplant Senior Program; KTs, kidney transplantations.

Supplementary Table 3. Short- and long-term outcomes of kidney transplants with and without AKI, stratified according to stages of donor AKI.

Characteristic	All KTs (n=685)	No AKI (n=502)	AKI stage 1 (n=151)	AKI stage 2+3 (n=32)	p-value
DGF	224 (32.8)	164 (32.8)	45 (29.8)	15 (46.9)	0.18
PNF	76 (11.1)	57 (11.4)	16 (10.6)	3 (9.4)	0.88
Length of hospital stay (days)	23.0 (16.0, 32.0)	22.0 (16.0, 34.0)	23.0 (16.0, 31.8)	25.0 (18.8, 32.0)	0.75
Recipient eGFR (ml/min/1,73 m²), median (IQR)					
3 months after transplant	32.7 (24.4, 43.2)	32.0 (24.0, 42.0)	35.0 (25.8, 45.0)	29.5 (23.0, 38.7)	0.13
1 year after transplant	35.0 (26.4, 44.5)	35.5 (26.3, 44.8)	34.0 (27.8, 45.2)	31.2 (25.0, 36.2)	0.11
3 years after transplant	35.0 (26.9, 47.7)	35.0 (26.6, 46.8)	37.0 (28.5, 53.4)	31.6 (23.0, 40.0)	0.08
5 years after transplant	36.0 (27.7, 48.1)	33.4 (23.7, 45.1)	40.5 (34.7, 49.4)	23.1 (19.0, 37.6)	0.21
BPAR in first year after KT	248 (38.6)	187 (39.3)	47 (34.6)	14 (46.7)	0.39
BPAR in first three years after KT	261 (40.7)	193 (40.5)	52 (38.2)	16 (53.3)	0.32
Death-censored graft survival					
1-year	80.5%	80.9%	78.3%	83.9%	
3-year	73.3%	72.8%	72.4%	83.9%	
5-year	67.4%	67.6%	67.6%	60.8%	
7-year	60.6%	61.3%	61.1%	41.7%	0.96
Overall graft survival					
1-year	74.8%	75.0%	74.0%	75.0%	
3-year	63.0%	62.0%	65.0%	68.0%	
5-year	54.8%	54.0%	58.3%	49.2%	
7-year	44.7%	42.9%	52.8%	30.0%	0.35
Patient survival					
1-year	88.9%	89.4%	89.1%	81.0%	
3-year	78.7%	78.3%	81.5%	73.4%	
5-year	72.8%	72.1%	74.9%	73.4%	
7-year	62.1%	60.2%	70.7%	47.2%	0.22
Follow-up time (months), median (IQR)	47.0 (24.0, 86.5)	44.0 (23.0, 86.0)	52.0 (23.0, 86.0)	47.0 (22.5, 67.5)	0.20

Numbers in brackets represent percentages if not indicated otherwise; percentages are based on the number of available cases for each parameter, excluding missing values; graft and- patient survival rates were calculated using the Kaplan-Meier-method; AKI, acute kidney injury; BPAR, biopsy proven acute rejection; DGF, delayed graft function; KTs, kidney transplantations; IQR, interquartile range; PNF, primary non function.

Supplementary Table 4. Incidence of BPAR within 3 years after KT stratified according to AKI-status and induction therapy.

AKI-status	BPAR (yes/no)	Basiliximab (n=566)	ATG (n=49)	none (n=23)
No-AKI	No	251 (44.3)	23 (46.9)	6 (26.1)
	Yes	171 (30.2)	7 (14.3)	14 (60.9)
AKI 1-3	No	88 (15.5)	8 (16.3)	2 (8.7)
	Yes	56 (9.8)	11 (22.4)	1 (4.3)

Numbers in brackets represent percentages; percentages are based on the number of available cases for each parameter, excluding missing values; AKI, acute kidney injury; ATG, Antithymoglobulin; BPAR, biopsy proven acute rejection.

Supplementary table 5. Short- and long-term outcomes of kidney transplants stratified by number of HLA-DR mismatches with and without donor AKI.

Characteristic	All KTs (n=685)	No AKI (n=502)	AKI (n=183)	p-value
BPAR in first year after KT				1.0
0 HLA-DR mismatches	31 (4.5)	23 (4.6)	8 (4.4)	
1 HLA-DR mismatch	95 (13.9)	71 (14.2)	24 (13.1)	
2 HLA-DR mismatches	120 (17.6)	91 (18.2)	29 (15.8)	
BPAR in first three years after KT				0.9
0 HLA-DR mismatches	33 (4.8)	24 (4.8)	9 (4.9)	
1 HLA-DR mismatch	98 (14.4)	73 (14.6)	25 (13.7)	
2 HLA-DR mismatches	125 (18.3)	94 (18.8)	31 (16.9)	
Death-censored graft survival				0.8†
1-year 0 HLA-DR mismatches	78.3%	74.8%	88.0%	0.2
1-year 1 HLA-DR mismatches	80.5%	80.6%	80.1%	1.0
1-year 2 HLA-DR mismatches	81.0%	83.0%	76.0%	0.2
7-year 0 HLA-DR mismatches	56.5%	57.0%	54.9%	0.6
7-year 1 HLA-DR mismatches	62.8%	62.9%	63.1%	0.8
7-year 2 HLA-DR mismatches	59.9%	61.2%	56.7%	0.4
Overall graft survival				0.3†
1-year 0 HLA-DR mismatches	77.3%	69.5%	84.0%	0.2
1-year 1 HLA-DR mismatches	74.9%	75.4%	73.6%	0.8
1-year 2 HLA-DR mismatches	74.8%	76.0%	71.8%	0.5
7-year 0 HLA-DR mismatches	45.9%	46.2%	46.3%	0.6
7-year 1 HLA-DR mismatches	45.4%	43.9%	49.6%	0.3
7-year 2 HLA-DR mismatches	43.4%	40.9%	50.6%	0.7
Patient survival				0.4†
1-year 0 HLA-DR mismatches	88.9%	87.8%	92.0%	0.6
1-year 1 HLA-DR mismatches	88.9%	90.0%	86.1%	0.4
1-year 2 HLA-DR mismatches	88.8%	89.2%	87.7%	0.8
7-year 0 HLA-DR mismatches	71.5%	72.5%	70.3%	1.0
7-year 1 HLA-DR mismatches	62.5%	61.5%	64.9%	0.7
7-year 2 HLA-DR mismatches	58.6%	55.5%	68.6%	0.4

Numbers in brackets represent percentages if not indicated otherwise; percentages are based on the number of available cases for each parameter, excluding missing values; graft and- patient survival rates were calculated using the Kaplan-Meier-method; AKI, acute kidney injury; BPAR, biopsy proven acute rejection; IQR, interquartile range; KTs,

kidney transplantations, p-values were calculated with Fither's Exact Test for count data and Log-Rank-Test for survival analysis,[†] Log-Rank-Test between no-AKI and AKI KTs stratified by number of HLA-DR mismatches for 7-year follow-up.

Supplementary Table 6. Short- and long-term outcomes of kidney transplants with and without donor AKI, stratified according to AKI recovery status.

Characteristic	All KTs (n=685)	No AKI (n=502)	AKI resolving (n=61)	AKI ongoing (n=122)	p-value
DGF	224 (32.8)	164 (32.8)	11 (18.0)	49 (40.2)	0.48
PNF	76 (11.1)	57 (11.4)	6 (9.8)	13 (10.7)	0.89
Length of hospital stay (days)	23.0 (16.0, 32.0)	22.0 (16.0, 34.0)	22.0 (16.0, 32.0)	23.0 (17.0, 32.0)	0.94
Recipient eGFR (ml/min/1,73 m²), median (IQR)					
3 months after transplant	32.7 (24.4, 43.2)	32.0 (24.0, 42.0)	38.7 (27.3, 44.6)	34.5 (24.6, 44.0)	0.39
1 year after transplant	35.0 (26.4, 44.5)	35.5 (26.3, 44.8)	36.4 (27.1, 42.8)	33.0 (27.3, 44.2)	0.87
3 years after transplant	35.0 (26.9, 47.7)	35.0 (26.6, 46.8)	37.3 (28.4, 51.3)	34.0 (28.0, 55.6)	0.54
5 years after transplant	36.0 (27.7, 48.1)	33.4 (23.7, 45.1)	34.7 (32.9, 39.3)	41.0 (33.5, 50.0)	0.48
BPAR in first year after KT	248 (38.6)	187 (39.3)	15 (24.6)	46 (37.7)	0.59
BPAR in first three years after KT	261 (40.7)	193 (40.5)	18 (41.9)	50 (40.7)	0.99
Death-censored graft survival					
1-year	80.5%	80.9%	80.0%	79.0%	
3-year	73.3%	72.8%	80.0%	72.3%	
5-year	67.4%	67.6%	66.8%	66.8%	
7-year	60.6%	61.3%	66.8%	56.9%	0.73
Overall graft survival					
1-year	74.8%	75.0%	80.0%	72.3%	
3-year	63.0%	62.0%	77.6%	61.5%	
5-year	54.8%	54.0%	62.4%	55.1%	
7-year	44.7%	42.9%	62.4%	46.0%	0.18
Patient survival					
1-year	88.9%	89.4%	91.0%	86.6%	
3-year	78.7%	78.3%	88.5%	77.2%	
5-year	72.8%	72.1%	85.5%	70.7%	
7-year	62.1%	60.2%	78.4%	63.6%	0.15
Follow-up time (months), median (IQR)	47.0 (24.0, 86.5)	44.0 (23.0, 86.0)	58.0 (42.0, 73.0)	48.0 (24.0, 94.0)	0.30

Numbers in brackets represent percentages if not indicated otherwise; percentages are based on the number of available cases for each parameter, excluding missing values; graft and- patient survival rates were calculated using the Kaplan-Meier-method; AKI, acute kidney injury; BPAR, biopsy proven acute rejection; DGF, delayed graft function; KTs, kidney transplantations; IQR, interquartile range; PNF, primary non function.

Supplementary Table 7. Fixed effects of multivariable Cox regression of death-censored graft loss and mortality of kidney transplant recipients.

Covariate	Death-censored graft loss		Mortality	
	**Hazard ratio (95% CI)**	**p-value**	**Hazard ratio (95% CI)**	**p-value**
Recipient age	1.02 (0.99-1.04)	0.15	1.07 (1.04-1.11)	<0.001*
Recipient sex: male vs. female	0.88 (0.66-1.17)	0.37	1.22 (0.89-1.69)	0.22
Recipient BMI	1.06 (1.03-1.10)	<0.001*	1.03 (0.99-1.07)	0.13
Duration of dialysis	1.01 (1.00-1.01)	0.01*	1.00 (1.00-1.01)	0.12
Recipient diabetes: yes vs. no	1.66 (1.18 -2.34)	<0.01*	1.55 (1.04 -2.30)	0.03*
Recipient arterial hypertension: yes vs. no	0.74 (0.49-1.12)	0.16	1.20 (0.68-2.12)	0.53
Highest PRA	1.00 (1.00-1.01)	0.56	1.01 (1.00-1.01)	0.14
Number of HLA mismatches	1.04 (0.94-1.15)	0.46	0.97 (0.86-1.08)	0.53
Number of KTs	1.46 (0.86-2.50)	0.16	1.48 (0.82-2.67)	0.19
Cold ischemia time	1.01 (0.98-1.05)	0.40	1.02 (0.98-1.07)	0.22
Donor age	1.01 (0.96-1.05)	0.77	1.03 (0.97-1.07)	0.45
Donor sex: male vs. female	1.02 (0.75-1.39)	0.90	0.74 (0.54-1.01)	0.06
Donor BMI	1.04 (1.00-1.09)	0.047*	1.01 (0.97-1.06)	0.65
Donor cause of death cerebral infarction: yes vs. no	1.36 (0.84-2.19)	0.21	0.80 (0.53-1.21)	0.29
Donor arterial hypertension: yes vs. no	1.01 (0.69-1.49)	0.96	1.48 (1.01-2.19)	0.047*
Donor diabetes: yes vs. no	0.64 (0.35-1.20)	0.16	1.21 (0.67-2.17)	0.53
Donor KDPI	1.01 (0.99-1.04)	0.22	0.99 (0.97-1.02)	0.64
Donor AKI: stage 1 vs. 0	0.92 (0.65-1.31)	0.66	0.75 (0.51-1.09)	0.13
Donor AKI: stage 2+3 vs.0	0.93 (0.46-1.87)	0.83	0.73 (0.93-3.25)	0.09

Missing values of co-variables: Recipient age: 0, Recipient sex: 0, Recipient BMI: 16, Duration of dialysis: 9, Recipient diabetes: 116, Recipient arterial hypertension: 116, Highest PRA: 11, Number of HLA mismatches: 3, Cold ischemia time: 46, Donor age: 0, Donor sex: 0, Donor BMI: 0, Donor cause of death cerebral infarction: 0, Donor arterial hypertension: 56, Donor diabetes: 90, Donor KDPI: 103, Donor AKI: 0.

AKI, acute kidney injury; BMI, body mass index; CI, confidence interval; CVA, cerebrovascular accident; HLA, human leucocyte antigen; KDPI, kidney donor profile index; KTs, kidney transplantations; PRA, panel reactive antibody; * if $p < 0.05$.

Supplementary Figure 1

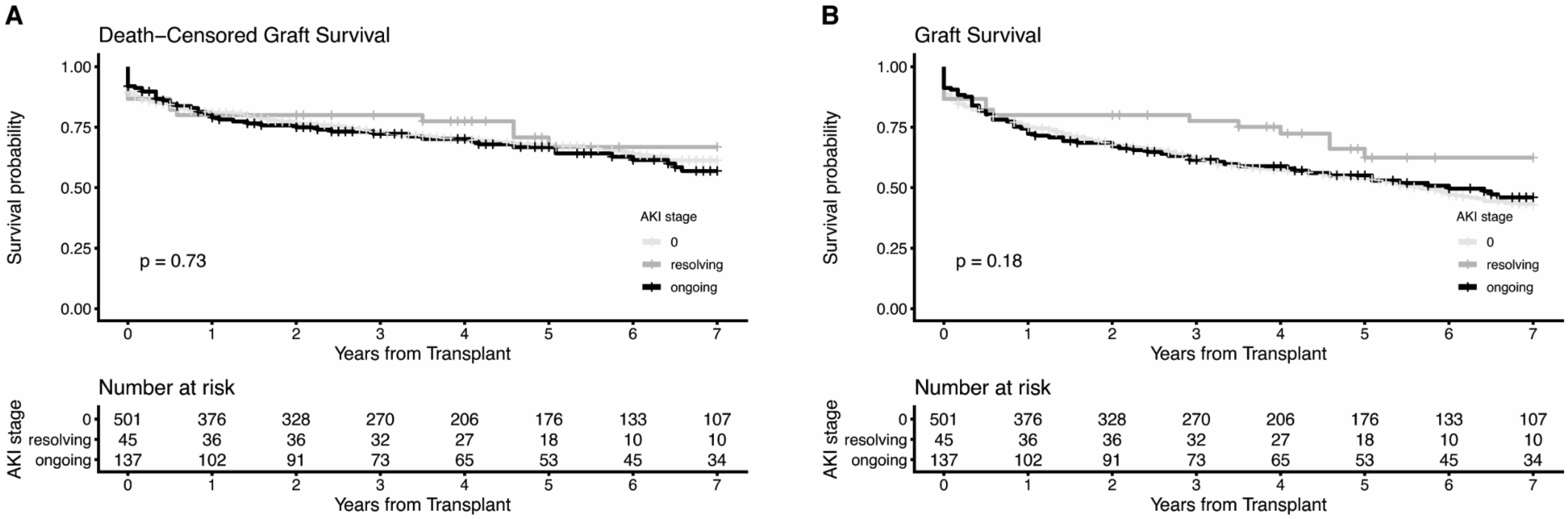

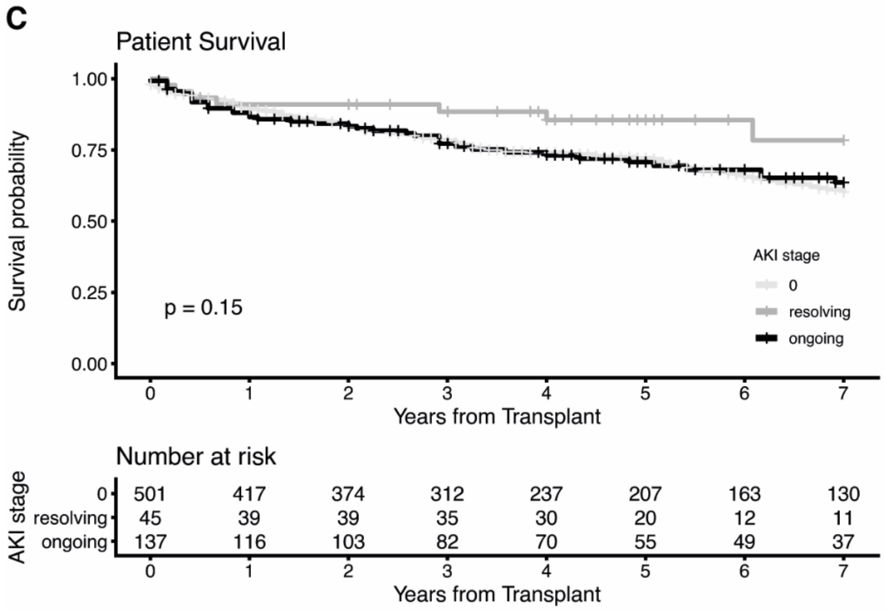

Figure legend supplementary Figure 1.

Kaplan-Meier estimates for death-censored graft survival (A), graft survival (B) and patient survival (C) of kidney transplants stratified by recovery status of donor acute kidney injury (AKI). The log-rank test was used to assess whether group distributions are equal.
